# An Account of a Contagious Fever, Which Occurred Amongst the Danish and American Prisoners of War, at Chatham, in the Years 1813, 1814

**Published:** 1831

**Authors:** 


An Account of a Contagious Fever, which occurred amongst the Danish and American Prisoners of War, at Chatham, in the Years 1813, 1814. By Sir William Burnett, Knt. M. D., K. C. H., Medical Commissioner of the Navy, &c. 8vo. pp. 47. Burgess & Hill, 1831.

The campaigns of Napoleon crowded the hospitals of the Continent with contagious fever, in the same way as those of the Russian Autocrat are now spreading pestilence and death through devoted Poland. Our insular situation and our wooden walls have secured us from these terrible attendants on hostile armies; but our naval ports have sometimes exhibited severe specimens of those afflictions, which are almost inseparable from war. Portsmouth, after Sir John Moore's retreat, was the scene of a desperate fever—and the prisoners of war, in all the different depôts, were occasionally scourged by fevers, which, however, usually sprang up among themselves from local causes, acquiring a contagious character in their course, and thereby aggravating their fatality. Of this kind was a fever which prevailed amongst the Danish and American prisoners of war on board several of the prison-ships in the Medway, in the Winter of 1813-14—and which Sir W. Burnett has now put on record, chiefly to shew the local origin and the contagious diffusion of the disease. The little work is also valuable in a prophylactic and therapeutic point of view, as the result of accurate observation and authentic histories.

Early in the month of Feb. 1814, a fever was reported to exist in the Bahama prison-ship, at Chatham; and Dr. Dickson, physician of the fleet, was directed to visit her. The number of cases then amounted to fifty among the prisoners, while some of the crew were also affected. It appears that in the preceding month (January) three or four hundred Danish prisoners had been sent from the Bahama to the Defiance, a temporary prison-ship — and in this *latter* ship a severe fever soon afterwards broke out. The disease advanced and increased in both vessels, and several deaths occurred. In the course of Feb. the fever appeared also in the Kron Prince, and Fyen prison-ships; but was of a milder character than in the two former vessels. On the 4th March Sir William was appointed to succeed Dr. Dickson, and found that, up to that time, 157 Danes and Americans had been sent to the hospital-ship, of whom 24 had died—and 21 afterwards. The hospital-ship was then extremely crowded, and many of the patients presented petechiæ, vibices, and gangrened legs. Mr. Thomson, the surgeon of the Bahama, had died of the fever, while his successor, Mr. Johnson, was ill of the same disease. To add to their embarrassment, the small-pox had broke out in the Kron Prince. It is remarkable, that the French prisoners in the surrounding prison-ships remained free from fever. The severity of the weather prevented the free ventilation of the infected ships; but every exertion was used to prevent the spread of the fever, by quickly separating the sick from those who were well. Towards the conclusion of March, the weather improved, and the fever gradually ceased. During the progress of the disease almost every medical officer, except the Doctor himself, suffered an attack of the fever. 518 men were affected after the 6th March, of whom 61 died—or about 1 in 8½. The disease made its appearance "with a surprising variety of symptoms, scarcely any two being attacked alike." The disease is minutely delineated by Sir William, but the symptomatology we must pass over. The morbid appearances, *post-mortem*, were as follow:—

### Brain.

"Vessels of the dura mater very turgid—meninges in different parts thickened and inflamed—coagulable lymph effused on the surface of the brain; and the substance of the brain itself much softened, in some cases accompanied with an effusion of blood.

### Thorax.

Lungs adhered to the pleura, and were often covered with pus. In some they were indurated; in others there were tubercles, or vomicæ, purulent effusion into the cavity of the thorax, and occasionally, effusion of blood. The fluid in the pericardium was often increased in quantity.

### Abdomen.

Concave surface of the liver, livid; and in one case, wherein the patient died suddenly while sitting by the fire in a state of apparent convalescence, there was encysted dropsy of that organ. The gall-bladder was much distended with inspissated bile, which could with difficulty be pressed through the duct into the duodenum.

The omentum and ileum were inflamed: the colon was found sometimes distended, at other times contracted and filled with fæces. The external parts of this intestine were in some instances of a greenish colour, with anasarcous effusion on its surface."

It can hardly be denied that the greater and more essential features of the foregoing phenomena must have resulted from inflammatory action, whether of the acute or sub-acute kind—and the antiphlogistic, when not obviously contra-indicated, was employed. "Would to God," says the author, "I could say that it was very successful. It was, however, the most successful of any we used." Some of the men when received into the hospital-ship, and who were covered with petechiæ, were (if the expression be allowed) "snatched from death by a moderate use of the lancet and purgatives, succeeded, in the decline of the disease, by a guarded employment of stimuli, such as wine and bottled porter." Mercury, as a sialogogue, was seldom useful — and emetics were rather injurious than beneficial. Much erysipelas prevailed, and a cold spirituous lotion was found to act almost as a specific.

### Etiology.

The Bahama was constituted a prison-ship in 1811; but it was only in 1814 that fever made any prominent appearance—and "increased alarmingly." It is quite evident that the Defiance, Kron Prince, and Fyen had had that kind of communication with the Bahama which led to the existence of the fever in those vessels. But how did the fever originate in the Bahama—the fons et origo of the evil? The weather, in the early part of the year 1814, was very inclement, in consequence of which the prisoners took "every method in their power to exclude the external air. They had, moreover, a great quantity of dirty clothes, and were exceedingly indolent—so that it was with the greatest difficulty that any thing approaching to a clean or well ventilated state of the prison-decks could be obtained." When these circumstances are taken into consideration, in conjunction with the depressed state of the *morale* among these people, "there seems just reason to conclude, that the fever in question originated among themselves from animal effluvia—became concentrated from the total want of ventilation — and that it afterwards assumed an infectious form, and propagated itself whenever there was sufficient communication with those infected." We have no doubt of it. It is the view which we have taken, since we were capable of forming a judgment from observation and reading. Sir William illustrates and confirms this view by instances collected from the journals of our naval medical officers in different quarters of the world, and which must carry conviction to every unprejudiced mind.

We have only been able to give a very brief analysis of this little volume, which we recommend to the perusal of all those who are sceptical as to the supervention of a contagious character on a fever originating in local causes.

